# Lactate secreted by glycolytic conjunctival melanoma cells attracts and polarizes macrophages to drive angiogenesis in zebrafish xenografts

**DOI:** 10.1007/s10456-024-09930-y

**Published:** 2024-06-06

**Authors:** Jie Yin, Gabriel Forn-Cuní, Akshaya Mahalakshmi Surendran, Bruno Lopes-Bastos, Niki Pouliopoulou, Martine J. Jager, Sylvia E Le Dévédec, Quanchi Chen, B. Ewa Snaar-Jagalska

**Affiliations:** 1 Institute of Biology, Leiden University, Leiden, 2333 BE The Netherlands; 2 Department of Ophthalmology, Leiden University Medical Center, Leiden, 2333 ZA the Netherlands; 3 Division of Drug Discovery and Safety, Leiden Academic Centre for Drug Research, Leiden University, Leiden, 2333 BE The Netherlands; 4 Division of Spine Surgery, Department of Orthopedic Surgery, Affiliated Hospital of Medical School, Nanjing Drum Tower Hospital, Nanjing University, Nanjing, 210008 China

**Keywords:** Angiogenesis, Conjunctival melanoma, Macrophages, Glycolysis, Lactate, Zebrafish model

## Abstract

Conjunctival melanoma (CoM) is a rare but potentially lethal cancer of the eye, with limited therapeutic option for metastases. A better understanding how primary CoM disseminate to form metastases is urgently needed in order to develop novel therapies. Previous studies indicated that primary CoM tumors express Vascular Endothelial Growth Factor (VEGF) and may recruit pro-tumorigenic M2-like macrophages. However, due to a lack of proper models, the expected role of angiogenesis in the metastatic dissemination of CoM is still unknown. We show that cells derived from two CoM cell lines induce a strong angiogenic response when xenografted in zebrafish larvae. CoM cells are highly glycolytic and secrete lactate, which recruits and polarizes human and zebrafish macrophages towards a M2-like phenotype. These macrophages elevate the levels of proangiogenic factors such as VEGF, TGF-β, and IL-10 in the tumor microenvironment to induce an angiogenic response towards the engrafted CoM cells in vivo. Chemical ablation of zebrafish macrophages or inhibition of glycolysis in CoM cells terminates this response, suggesting that attraction of lactate-dependent macrophages into engrafted CoM cells drives angiogenesis and serves as a possible dissemination mechanism for glycolytic CoM cells.

**Supplementary Information:**

The online version contains supplementary material available at 10.1007/s10456-024-09930-y.

## Introduction

Conjunctival melanoma (CoM) arises from melanocytes located amongst the basal cells of the conjunctival epithelium. CoM can appear on any part of the conjunctiva and spreads via lymphatic as well as hematogenic routes [1]. CoM is a rare but potentially malignant cancer, prevalent in Caucasians, with a rising incidence in recent decades. CoM currently affects 0.3 to 0.8 individuals per million [2]. Tumor recurrence is estimated to be around 65% at 15 years, and distant metastases develop in about 30% of patients, and are usually located in the lung, liver, skin, or brain [3]. Once metastasized, the survival of the majority of CoM patients is less than 10 years [4]. Current therapeutics for primary CoM are effective, but treatment options after CoM has metastasized are limited [5]. Therefore, it is necessary to identify the mechanisms of metastatic dissemination to develop possible new interventions to prevent metastasis and to treat metastasized CoM.

A major process governing tumor growth, dissemination and metastasis is the ‘angiogenic switch’, where a balance between pro-angiogenic and anti-angiogenic factors shifts towards angiogenesis and helps the conversion of a benign tumor into a malignant state. Angiogenesis is often regarded as a precursor to tumor metastasis and considered as one of the hallmarks of cancer [6]. Most solid tumors require vasculature to deliver enough oxygen, nutrients, and for waste disposal, thus making angiogenic processes mandatory to sustain proliferation of tumor cells [7]. During angiogenesis, primary tumors stimulate the “sprouting” of novel endothelial cells from surrounding pre-existing blood cells, thus expanding the vascular tree [8]. There are several known pro-angiogenic factors that trigger angiogenesis, including vascular endothelial growth factor (VEGF), epidermal growth factor (EGF), transforming growth factor-α and -β (TGF-α and -β), interleukin 10 (IL-10) and angiopoietin 1 and 2 (Ang-1 and − 2). The VEGF family is often considered essential [9].

Primary CoM is characterized by hypovascular tumors, even though VEGF immunoreactivity was detected in the tumor tissue [10]. Two CoM cell lines (CRMM1, CRMM2), both derived from a local recurrence, also express VEGFs, which affected migration and proliferation of endothelial cells [11]. Another study revealed that adjuvant anti-VEGF therapy contributed to the improvement of disease-free survival in CoM patients with a high risk of recurrence [12]. It remains elusive whether VEGF-driven angiogenesis affects CoM growth and metastatic dissemination in vivo.

The source of cytokines and growth factors that regulate angiogenesis are often immune cells in the tumor microenvironment (TME), such as macrophages [13]. Macrophages that reside in tumors are known as Tumor Associated Macrophages (TAMs), and are traditionally categorized into two subsets: M1 and M2 [14]. M1 macrophages secrete many cytokines, including interleukin 1-beta (IL-1β), tumor necrosis factor alpha and beta (TNF-α and TNF-β), and interleukin 6 (IL-6), and are regarded as anti-tumor or pro-inflammatory, while M2 are considered as pro-tumor or anti-inflammatory [15]. It has been reported that M2 type TAMs participate in tumor angiogenesis by secreting pro-angiogenic factors, including VEGF-A, TGF-β, and C-C motif chemokine ligand 2 (CCL2) [16].

M2-type macrophages have been linked to increased tumor angiogenesis in uveal and cutaneous melanomas [17–20]. Unfortunately, the role of macrophages and other infiltrated lymphocytes in CoM is still debated. Some studies indicate no correlation between the presence of infiltrate and CoM prognosis, while others report an association between a lack of inflammatory cells and poor prognosis [21]. In 2017, Cao et al., analyzed 27 primary CoM patient samples by immunofluorescence, and all samples showed infiltrating macrophages, albeit in varying amounts. Especially CD68 and CD163 positive M2 macrophages were observed, but the amount of M2 macrophages did not significantly correlate with patient survival or recurrence [20].

Warburg observed that cancer cells preferentially perform glycolysis by converting glucose molecules into lactate regardless of the amount of oxygen present, a process known as aerobic glycolysis [22]. During glycolysis, cells take up glucose via the glucose transporter 1 (GLUT1) and convert glucose into pyruvate through ten consecutive enzymatic reactions in an oxygen-independent manner. Then, newly generated pyruvate is either catalyzed into acetyl-CoA to fuel the tricarboxylic acid (TCA) cycle in a slow but efficient process that requires oxygen, or is rapidly reduced to lactate by the enzyme lactate dehydrogenase (LDH) under anaerobic conditions. During aerobic glycolysis, cancer cells produce lactate even in the presence of oxygen, and intracellular excess of lactate is released from cells by monocarboxylate transporters (MCTs) [23]. Cellular lactate levels have been used to evaluate the metabolism of cancer due to the importance of lactate to cancer growth. Aerobic glycolysis is not only a potent fuel to promote tumor cell growth, but lactate is also a signaling molecule in the tumor-micro-environment (TME): lactate accumulation is linked to immune suppressive phenotypes of tumor-infiltrating immune cells [24].

In 2014, Colegio and colleagues discovered that tumor-derived lactate is a key factor that functionally polarizes TAMs [25]. They showed in three different syngeneic tumor xenograft models, including murine B16 cutaneous melanoma cells, that lactate serves as a communication signal between macrophages and tumor cells. Tumor-derived lactate induced the M2-like polarization of TAMs through the induction of arginase 1 (Arg-1) expression via the hypoxia-inducible factor 1α (HIF1α) – VEGF pathway. It was generally assumed that cancer cells experiencing hypoxia in a growing tumor are themselves the source of VEGF [26], but the findings of Colegio et al., suggest that, at least in some cases, it is the tumor accessory cells, including macrophages, that are the main source of VEGF. We did not find studies analyzing the metabolism of CoM, but considering its genetic similarity to cutaneous melanoma [27, 28], it can be anticipated that CoM cells also produce lactate even in the presence of oxygen.

It is not clear in CoM whether vessel development, a potential avenue for metastatic dissemination, is regulated by the expression of VEGF by the tumor cells themselves or by M2-polarized infiltrating macrophages. In order to clarify the relationships between TAM, angiogenesis, and tumor metabolism, we utilized well-described tumor angiogenesis assay using zebrafish larvae [29]. The optical transparency of zebrafish embryos together with the use of the transgenic tumor cell lines provides a reproducible xenograft model with fluorescent vessels and macrophages, which allows live imaging of tumor cells, macrophage recruitment, and the subsequent angiogenetic response. Previous research demonstrated that this angiogenetic model represents a novel tool for investigating the neovascularization process exploitable for drug discovery and gene targeting in tumor angiogenesis. The use of zebrafish xenografts models [29] allows the continuous delivery of angiogenic factors produced by a limited number of tumor cells, thus mimicking the initial stages of tumor angiogenesis and metastasis.

To investigate the role of angiogenesis and metabolism, we engrafted CoM cells into the perivitelline space (PVS) of transgenic zebrafish embryos. This model allows simultaneous live imaging of engrafted cells, macrophage recruitment and the angiogenetic response. Next to two CoM cell lines (CRMM1, CRMM2), we also engrafted as reference two isogenic breast cancer cell lines (highly metastatic and glycolytic 4T1 and non-metastatic, non-glycolytic 67NR) [30]. We observed that both CoM cell lines and the highly glycolytic 4T1 cells induced macrophage-dependent angiogenesis. Chemical ablation of macrophages reduced the angiogenic response to glycolytic cancer cells. Also, the pharmacological inhibition of glycolysis prior to engraftment lowered production of lactate by cancer cells, and diminished macrophage recruitment and their polarization, leading to an inhibition of angiogenesis. Our results suggest that CoM cell lines (CRMM1, CRMM2) are highly glycolytic and secrete lactate for the recruitment and polarization macrophages to a M2-like type. The increased expression of VEGF and other pro-angiogenetic factors from these macrophages in the proximity of the engrafted cells then induces an angiogenic response, which is required for the metastatic dissemination.

## Materials and methods

### Cell culture

Human conjunctival melanoma cell lines CRMM1 and CRMM2 [31] were cultured in F12 Kaighn’s modified medium (Hyclone, cat# SH30526.01) supplemented with 10% fetal bovine serum (FBS; Gibco). Breast cancer 67NR and 4T1 (ATCC, USA) were grown in Dulbecco’s modified Eagle’s medium (Hyclone, cat# SH30526.01) with 10% fetal calf serum. All cell lines were maintained in a humidified incubator at 37 °C with 5% CO2. To enable visual tracking in zebrafish xenografts, cells were transduced with lentivirus expressing mCardinal (far-red) and selected with puromycin.

### Zebrafish handling

We used the zebrafish transgenic line Tg(*kdrl*:EGFP^s843^; *mpeg1*:GAL4-VP16^gl24^; UAS-E1b: NfsB-mCherry^i149^). All animal experiments were conducted in compliance with local animal welfare regulations and handled according to standard protocols (www.ZFIN.org). Zebrafish embryos were collected and maintained in egg water at 28.5 °C. Zebrafish larvae were anaesthetized using 0.003% Tricaine (Sigma) for engraftment and imaging.

### Zebrafish engraftment and angiogenesis quantification

Prior to injection, cultured cells were disassociated using trypsin and centrifuged at 1000 rpm for 5 min. The pellet was resuspended in 2% Polyvinylpyrrolidone 40 (PVP; Sigma-Aldrich) in PBS at a final concentration range of 250–500 × 10^6^ cells/mL. The cell suspension was loaded in glass capillary needles (1.0 mm O.D. x 0.78 mm I.D.; Harvard Apparatus) and 100–200 cells or the same volume of 2% PVP were injected in the PVS per larvae using a Pneumatic Pico pump (World Precision Instruments). Xenografts were screened 2 h post injection and maintained in egg water at 33 °C for the remainder of the experiment. For macrophage ablation studies, metronidazole (MTZ) was added to the egg water 2 h post engraftment.

After 24 h, zebrafish larvae were anaesthetized and imaged using a Leica TCS SP8 confocal microscope with a 20X objective equipped with 488-nm, 532-nm, and 638-nm laser lines. For image quantification, the SIV length was defined by the sum lengths of all blood vessels. The SIV angle is defined by the angle between the two most outward veins of the SIV. The elongation of the SIV complex is calculated by dividing the length of the SIV complex by its width. For timelapse experiments, live engrafted larvae were mounted in 1% low melting agarose (SERVA, 140,727) in E3 medium containing 0.03% tricaine.

### Real time quantitative PCR (qPCR)

Engrafted embryos (15 embryos per group, three independent replicates) were anaesthetized and collected in TRIzol (Invitrogen) and homogenized. RNA was isolated using the chloroform / isopropanol method and resuspended in Nuclease-free water. Total RNA was quantified using Nanodrop. cDNA was prepared from RNA (100ng/µL) using QUANTA Biosciences qScriptTM cDNA Supermix following the manufacturer’s protocol. The resulting cDNA was used for quantifying gene expression using a Bio-Rad qPCR kit with primers indicated in Supplementary Tables 2 and analyzed using the ΔΔCT method.

### SRB assay

For the SRB assay, cells were seeded in a 96-well plate. The next day, specified concentrations of 2DG or GSK2837808A treatment were added to the medium. Cells were fixed after 24 h of treatment using 30µL 50% Trichloroacetic acid (TCA, Sigma-Aldrich) for 1 h at 4 °C. Cellular proteins were stained with 0.4% SRB (Sigma-Aldrich) for 30 min and washed with 1% acetic acid (VWR, Amsterdam, The Netherlands) to remove the unbound SRB. Protein-bound SRB was dissolved in 200µL 10mM in buffered Tris (Thermo Fisher Scientific) and absorbance was measured at 540 nm on M1000 microplate reader (Tecan, Giessen, The Netherlands).

### ATP luciferase assay

For ATP quantification, cells were seeded on black screen-star plates (Greiner, the Netherlands) and incubated overnight according to the protocol for ATPlite 1 step Kit (PerkinElmer, the Netherlands). Cells were stained with Hoechst for 45 min and images of nuclei were captured by Nikon Eclipse Microscope using Plain Fluor10 objective. At the time of measurement, each well in the plate was replaced with 50µL fresh media after which 50µL of ATP substrate was added. The plate was subjected to shaking for 2 min and luminescence was quantified using FLUO star plate reader (BMG Labtech, the Netherlands). Shown values are normalized to the nuclei fluorescence as a proxy for the number of cells in the well.

### Lactate assay

Cancer cells were cultured in a 96-well plate with 100 µL of medium. At the time of quantification, supernatants were transferred to a new plate. 10 µL of the supernatant was added to 90µL of the working lactate assay reagent (108mM Triethanolamine HCl, 10.7mM EDTA.Na_2_, 42mM MgCl_2_). The mixture was incubated in the dark for 7 min at room temperature and absorbance was measured at 490 nm using FLUO star plate reader (BMG Labtech, the Netherlands). Shown values are normalized to the number of cells in the well.

### Treatment of macrophages with conditioned medium

CRMM1 and CRMM2 cells were seeded into 12-well plates and treated with 10mM of 2DG, 10µM GSK2837808A or the same volume of DMSO for 24 h, when the medium was refreshed and kept for an additional 24 h. Human monocytes (THP-1 cell line) were seeded into a 12-well plate and differentiated into macrophages using phorbol 12-myristate 13-acetate (PMA) for 24 h. Differentiated M0-like macrophages were then divided and treated with supernatant medium from the CRMM1 and CRMM2 cultures treated with 2DG, GSK2837808A or DMSO, 10 μm of lactate as a positive control, or the same volume of DMSO as a negative control. After 24 h of exposure, macrophages were collected for RNA extraction and qPCR analysis.

### Statistical analysis

All experiments were performed at least 3 times with independent biological replicates, including at least 15 embryos per group. Error graphs shown in the figures represent the SEM. Statistical significance was established by one-way ANOVA and plotted using GraphPad Prism 9.1. For plotting, * indicates 0.01 < *p* < 0.05; ** indicates 0.001 < *p* < 0.01, *** indicates 0.0001 < *p* < 0.001. **** indicates *p* < 0.0001.

## Results

### Metastatic conjunctival melanoma cells induce angiogenesis and recruitment of macrophages in a zebrafish model

**Fig. 1:**
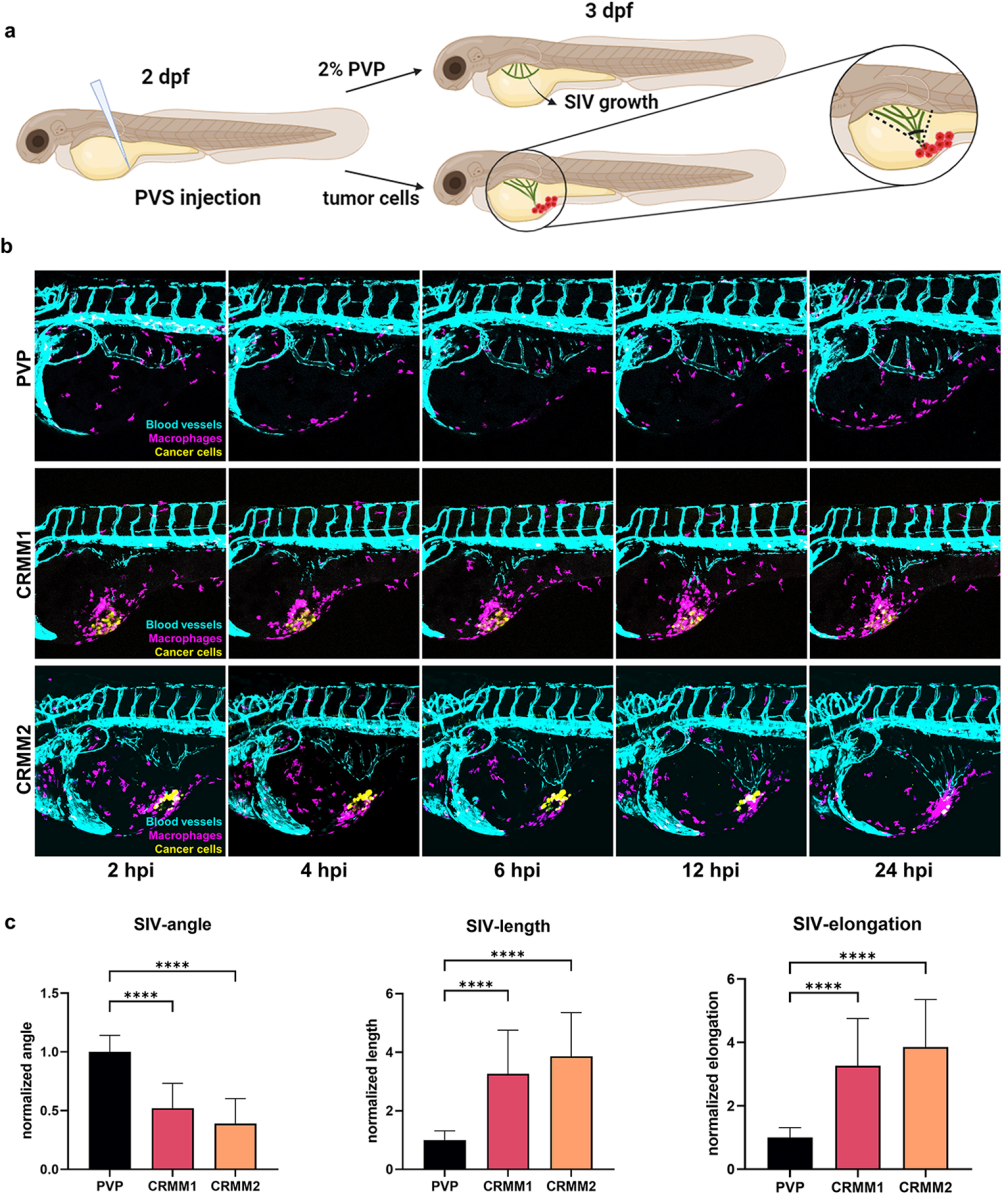
Metastatic conjunctival melanoma cells induce angiogenesis and recruitment of macrophages in a zebrafish angiogenesis model. **a** Schematic representation of the angiogenesis assay in zebrafish xenografts. Cancer cells or 2% PVP used as vehicle are injected into the PVS at 2 day post fertilization (dpf) zebrafish larvae Tg(*kdrl*:EGFP^s843^; *mpeg1*:GAL4-VP16^gl24^; UAS-E1b: NfsB-mCherry^i149^). In response to a strong angiogenic signal, the SIV complex is deformed towards the injection focus. **b** Representative images of time-lapses recording the angiogenic response to the SIV from 2 to 24 hpi. **c** Quantifications of the angiogenic activity induced by engraftment of CoM cells at 24hpi as measured by the angle, total length, and elongation of the SIV complex

To explore if conjunctival melanoma cells can trigger macrophage-dependent angiogenesis, we applied a well-described zebrafish tumor xenograft angiogenesis assay, which allows imaging of angiogenetic activity after 16–24 h [29]. In this assay, the growth of the blood vessels from the sub-intestinal vein plexus (SIV) is induced by the engraftment of tumor cells, triggering sprouting or remodeling of the SIV complex without adverse effects on other physiological vessels (Fig. 1a, Supplementary Movie 1). For this purpose, we injected the CoM cell lines CRMM1 and CRMM2 (both derived from a local recurrence) stably labelled with mCardinal (far-red fluorescence) into the perivitelline space (PVS) of Tg(*kdrl*:EGFP^s843^; *mpeg1*:GAL4-VP16^gl24^; UAS-E1b: NfsB-mCherry^i149^) zebrafish embryos. This zebrafish transgenic line expresses the green fluorescence protein (EGFP) under the promoter of the kinase insert domain receptor (*kdrl*, zebrafish homolog of VEGF receptor) [32], which labels endothelial cells, and the fusion protein of *E. coli* NsfB reductase and mCherry under the macrophage-expressed gene 1.1 (*mpeg1.1*), thus labelling macrophages. The kinetics of the induced angiogenic response in live embryos was monitored directly after engraftment by time-lapse imaging from 2 h post injection (hpi) to 24 hpi in 15 min intervals. As shown in Fig. 1b, there was a fast recruitment of macrophages to the position of engrafted cells (~ 2hpi) and a pronounced colocalization with cancer cells during the first 24hpi, coinciding with the elongation of the SIV towards the injection foci.

To quantify the angiogenic activity induced by engraftment of CoM at 24hpi, the angle, total length, and elongation of the SIV complex were measured (Fig. 1c). Both CRMM1 and CRMM2 significantly decreased the directional angle and increased SIV length and elongation, illustrating the induction of angiogenesis. In comparison, the angle, length, and elongation of the SIV complex of zebrafish injected with vehicle without cells showed no modification despite the initial recruitment of macrophages, which was considered a transient inflammatory reaction to wounding.

In conclusion, these results demonstrate that both of the CRMM1 and CRMM2 cell lines have a high angiogenic activity in the zebrafish xenograft model, correlating with macrophage recruitment.

### The angiogenic response induced by engraftment of metastatic CoM cells is macrophage-dependent

To study the functional significance of macrophage recruitment towards engrafted CoM cells during the development of angiogenesis, we used the NsfB nitroreductase (NTR) / Metronidazole (MTZ) ablation system to deplete macrophages in living zebrafish embryos. The zebrafish transgenic larvae mentioned above express NTR in their macrophage lineage. Upon delivery of MTZ in the culture medium, NTR converts this substrate into a cytotoxic agent capable of killing macrophages [33]. First, we optimized the efficiency of macrophage ablation without signs of toxicity. Embryos from 2dpf until 8dpf were exposed to various concentrations of MTZ (Supplementary Fig. 1a). Embryos treated with 10mM MTZ showed higher lethality than controls, indicating that MTZ was toxic at higher concentrations, but lower concentrations had no effect on the survival of embryos. We concluded that 2.5mM MTZ successfully depleted macrophages in embryos from 2dpf to 5dpf without adverse effects and used this concentration to examine to role of macrophages in tumor-induced angiogenesis (Supplementary Fig. 1b and c).

**Fig. 2:**
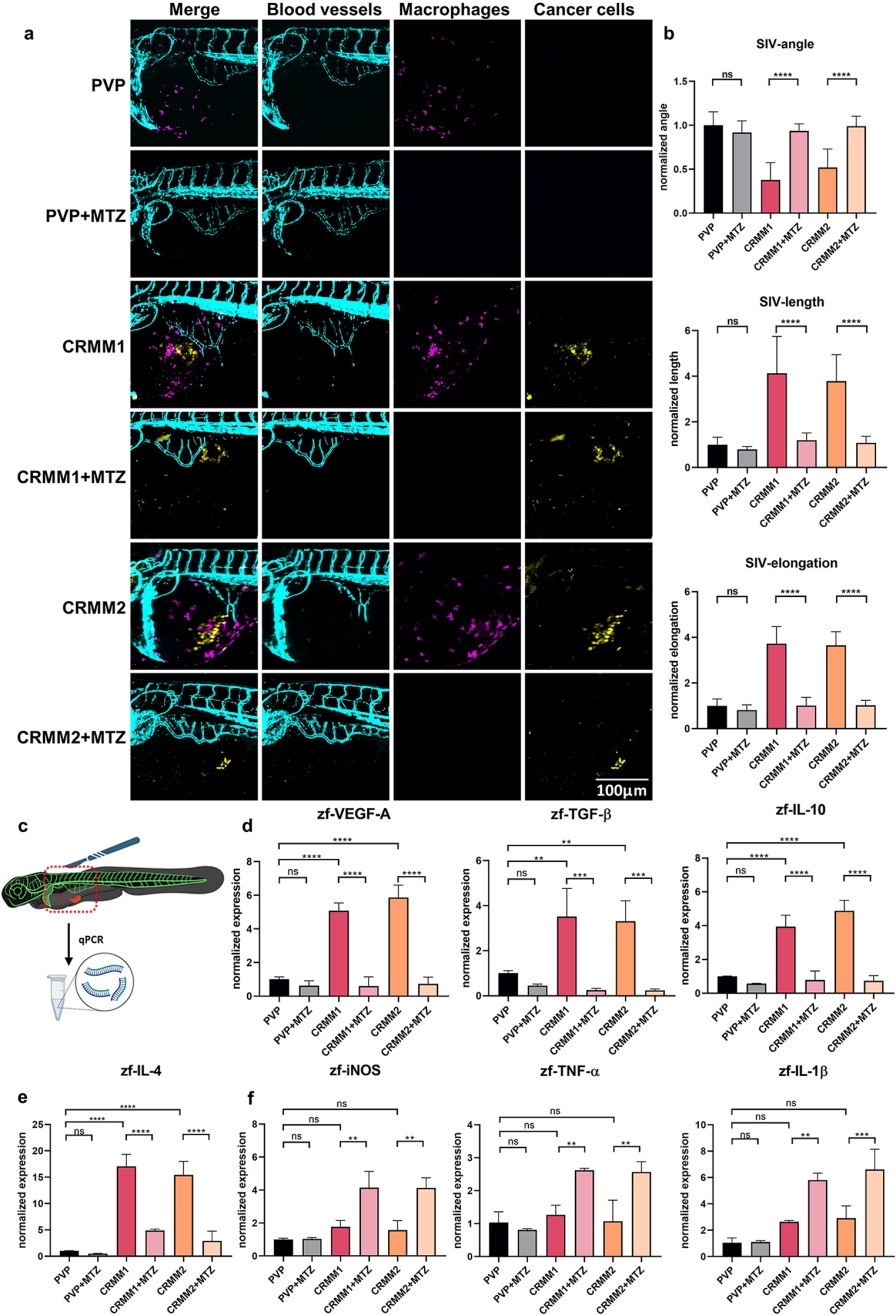
Chemical ablation of macrophages inhibits tumor-induced angiogenesis in a zebrafish model. **a** Representative images of xenografted zebrafish larvae, treated with or without 2.5mM MTZ at 24hpi. **b** Quantification of the angiogenic capacity of cancer cells with and without macrophages. **c** Xenografted zebrafish material was retrieved and used to detect angiogenetic and inflammatory factors with qPCR. **d** Expression levels of zebrafish VEGF-A (*vegfaa*), TGF-β (*tgfb1a*), and IL-10 (*il10*) in CRMM1 and CRMM2 engrafted groups. **e** Expression of the M2 marker IL-4 (*il4*) in tumor engrafted zebrafish treated with DMSO and MTZ. **f** Expression of proinflammatory markers, iNOS (*nos2a*), IL-1β (*il1b*), and TNF-α (*tnfa*) in DMSO or MTZ-treated groups

To this aim, we injected embryos with the 2% PVP-containing vehicle as control or mCardinal-labeled CRMM1 and CRMM2 cells at 2dpf and treated the embryos with 2.5mM TMZ from 2hpi. Under these conditions, we imaged and quantified the angiogenetic activity at 24hpi (Fig. 2a and b). In the PVP-injected group, the developmental pattern of SIV was unchanged regardless of the presence or absence of macrophages. As described in Fig. 1b and c, engraftment of CoM cells lines induced macrophage recruitment and angiogenesis, but chemical ablation of macrophages by MTZ resulted in reduction of the angiogenetic response as quantified by measurement of the SIV directional angle, length, and elongation (Fig. 2b). Thus, the presence of macrophages is essential for the induction of angiogenesis by the conjunctival melanoma cell lines CRMM1 and CRMM2 in our model. Importantly, MTZ treatment of wild type embryos (without NTR) engrafted with CRMM1 and CRMM2 cells had no effect on the tumor cell viability and tumor-induced angiogenesis suggesting that MTZ operates strictly via NTR-dependent ablation of macrophages (Supplementary Fig. 1d).

To further explore if zebrafish macrophages recruited to CRMM1 and CRMM2 induced tumors polarized to an M2-like phenotype, which is associated with angiogenesis in mice [34], the expression level of pro-angiogenic, pro-inflammatory, and M2 markers in engrafted zebrafish larvae were quantified by qPCR using zebrafish-specific primers (Fig. 2c-f). We found that pro-angiogenic factors, such as VEGF-A, TGF-β, and IL-10 were overexpressed in the groups injected with CRMM1 and CRMM2 compared to the PVP vehicle controls, but that the expression of these genes was reduced if macrophages were ablated (Fig. 2d). The same pattern was observed with the M2 marker IL-4 (Fig. 2e). In contrast, the expression level of the proinflammatory markers iNOS, TNF-α, and iL-1β was increased in the absence of macrophages (Fig. 2f).

Altogether, these results suggest that angiogenesis caused by the engraftment of metastatic conjunctival melanoma cells is macrophage-dependent, and that recruited macrophages are polarized towards a M2-like stage in the proximity of tumor cells.

### Lactate produced by glycolytic cancer cells attracts and polarizes zebrafish macrophages to promote angiogenesis

**Fig. 3:**
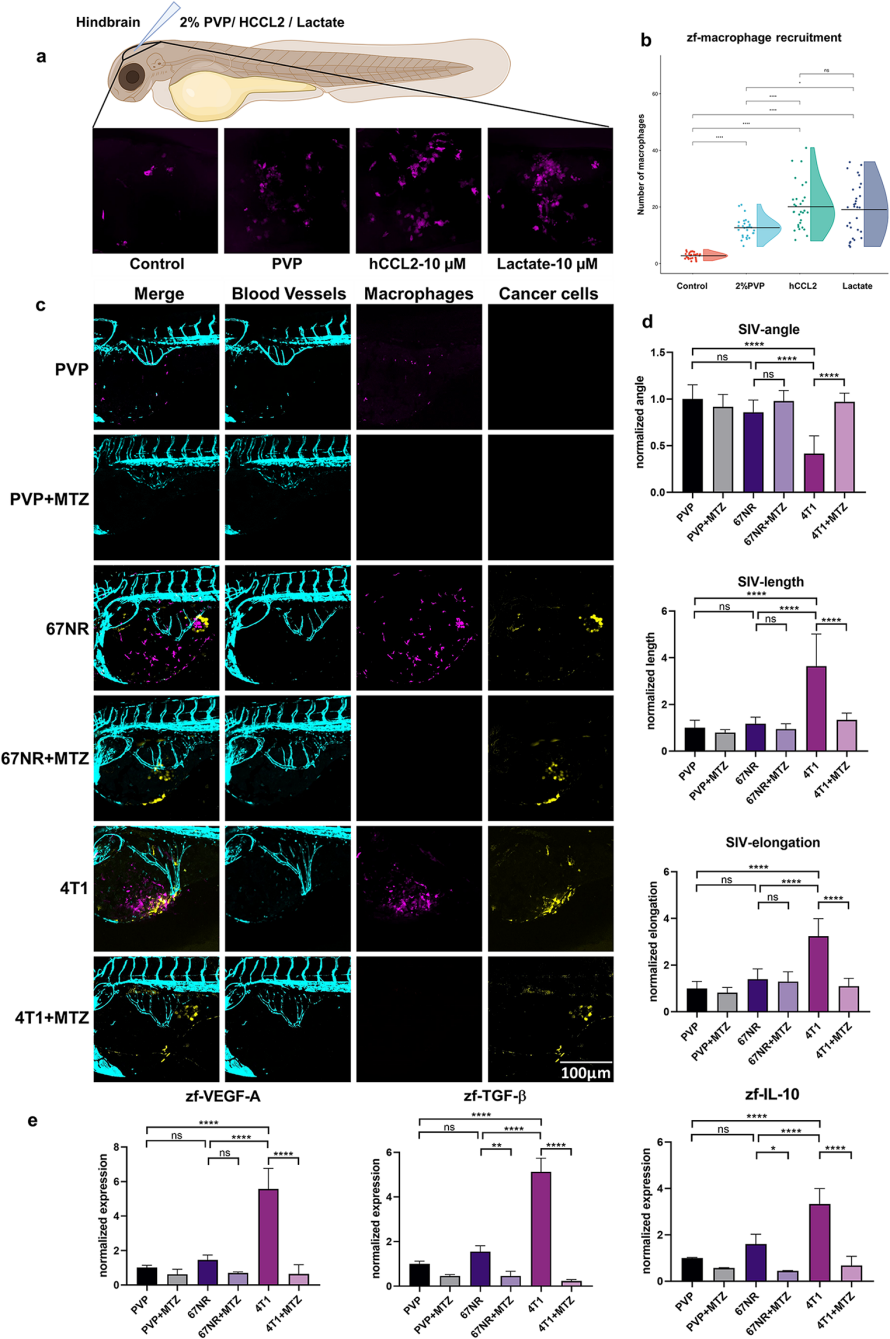
Lactate and glycolytic 4T1 cells attract zebrafish macrophages. **a** Schematic diagram of injection of lactate and hCCL2 into the zebrafish hindbrain. **b** Quantification of the macrophages attracted to the hindbrain under different conditions. **c** Representative images of the angiogenic effect in the SIV complex with and without treatment with MTZ at 24 hpi. **d** Quantification of the angiogenic influence of the 67NR and 4T1 cell lines with and without macrophages. **e** Expression of the zebrafish-derived proangiogenic markers VEGF-A (*vegfaa*), TGF-β (*tgfb1a*), and IL-10 (*il10*)

We then wondered how macrophages are recruited and retained to the site of CoM cell engraftment. We hypothesized that, as initially described by Colegio et al. [25], lactate produced by glycolysis of cancer cells attracts macrophages to facilitate the angiogenetic response. In order to explore this possibility, we first studied if zebrafish macrophages are able to sense lactate as demonstrated for mammalian macrophages. Analysis of the zebrafish single cell atlas from the Miller lab [35] reveled that indeed paralogs of human lactate receptors GPR81 and GPR132 are expressed on zebrafish macrophages (Supplementary Fig. 2 and Table 1). To test this functionally, we directly injected lactate into the hindbrain of 2dpf zebrafish transgenic larvae with fluorescently labelled macrophages, which is free of this cell type at this developmental stage (Fig. 3a). The human cytokine CCL2, a well-known chemoattractant of zebrafish macrophages [36], was used as positive control, while we used PVP solvent to determine the macrophage influx to the local inflammation generated by the injection wound. Injection of CCL2 and lactate significantly increased the number of accumulated macrophages in the hindbrain 3hpi compared to the number of macrophages in the same area of control and PVP-injected embryos, demonstrating that indeed lactate can drive zebrafish macrophage migration (Fig. 3b).

Next, we tested whether highly glycolytic malignant cells, which preferentially metabolize glucose to lactate even in aerobic conditions, can efficiently attract zebrafish macrophages in vivo. For this, we used two reference isogenic breast cancer cell lines: the highly metastatic, glycolytic 4T1, and the non-metastatic, low-glycolytic 67NR [30]. It has previously been reported that 4T1 cells generate 10-fold more lactate than isogenic non-metastatic 67NR cells [37]. Engraftment of control PVP vehicle and of 67NR cells did not modify SIV growth (Fig. 3c and d). In contrast, the isogenic metastatic variant 4T1 caused a significant angiogenetic response and recruited more macrophages compared to the vehicle PVP control and to 67NR cells. Similar to the CRMM1 and CRMM2 lines, the angiogenetic effect of the engrafted 4T1 line was dependent on macrophages, which were required for the expression of zebrafish angiogenic factors.

The differential angiogenetic phenotype seen by the engraftment of 4T1 and 67NR cells in the zebrafish model suggests that tumor-derived lactate can drive macrophage recruitment and the angiogenic response in this zebrafish angiogenesis model. In addition, we show the capacity of the zebrafish short-term angiogenesis assay to discriminate between highly angiogenic and poorly angiogenic tumor cell lines, thus allowing a critical examination of the angiogenetic properties of the CRMM1 and CRMM2 cell lines.

### Conjunctival melanoma cells secrete lactate and maintain their glycolytic properties after xenografting

**Fig. 4:**
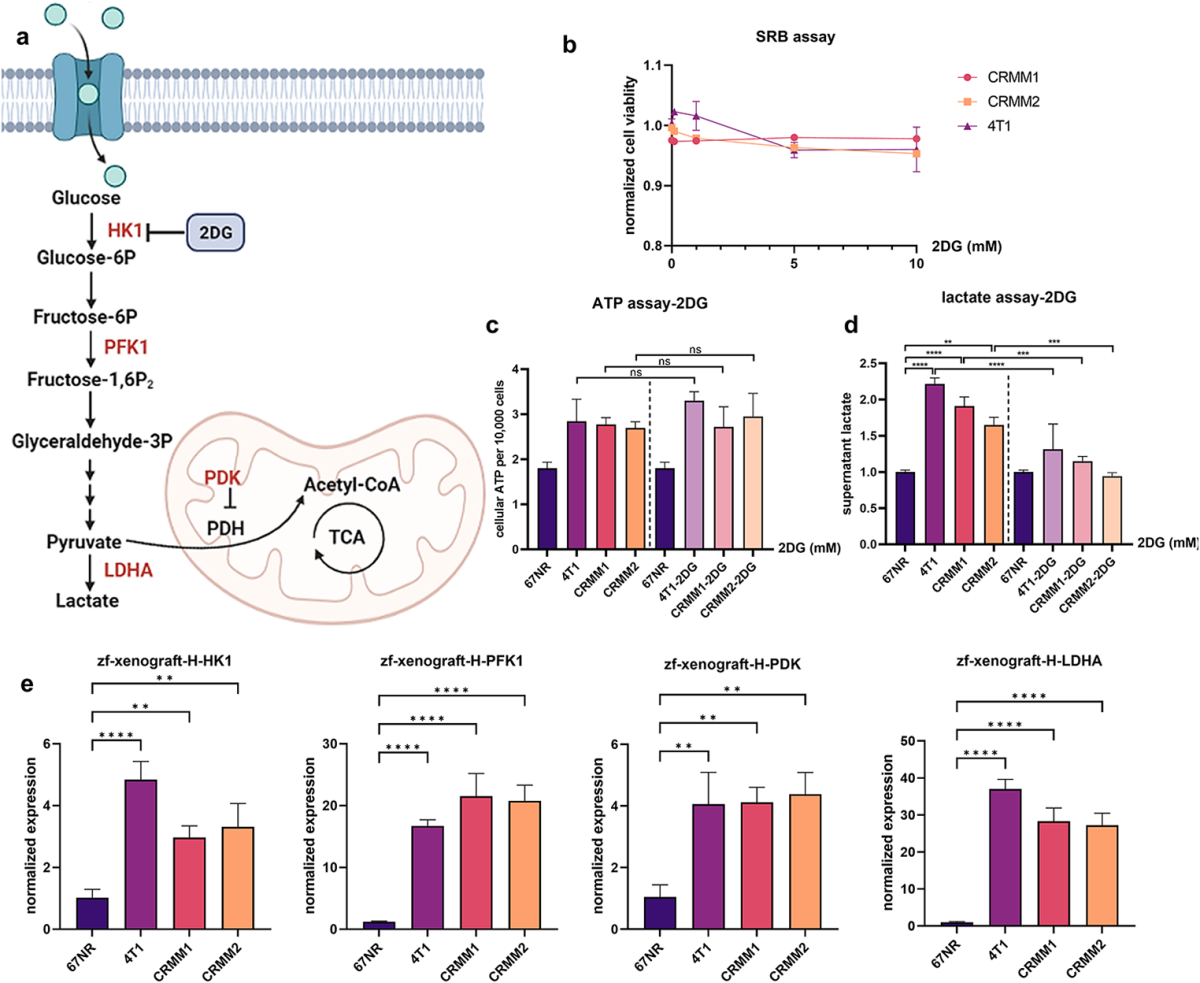
CoM cells secrete lactate and maintain their glycolytic properties in xenograft models. **a** Schematic representation of the glycolysis pathway with key enzymes in the process, showing the effect of 2DG inhibiting the activity of Hexokinase 1 (HK1). **b** Quantification of cell viability during treatment of increasing 2DG concentrations for 24 h. **c** The cellular ATP level of 67NR and 4T1, CRMM1, and CRMM2 after 2DG treatment. **d** 2DG inhibited the lactate production in 67NR and 4T1, CRMM1, and CRMM2 cells. **e** Expression of the glycolysis-related enzymes HK1, PFK1, PDK, and LDHA in zebrafish 4T1, 67NR, CRMM1 and CRMM2 xenografts

Given the possible effect of lactate on angiogenesis, we questioned whether CRMM1 and CRMM2 cells also secrete lactate to govern macrophage recruitment and polarization leading to induction of angiogenesis in the zebrafish model. To evaluate the glycolytic properties of CRMM1 and CRMM2, the level of lactate was measured in their supernatant with and without pretreatment with 2-Deoxy-D-glucose (2DG) and with GSK2837808A (GSK), and compared to lactate level in the reference low-glycolytic 67NR and high glycolytic 4T1 cell lines (Fig. 4a-d, Supplementary Fig. 3a-d). 2DG blocks glycolysis by competitively inhibiting Hexokinase 1 (HK1), an enzyme responsible for the phosphorylation of glucose in the first step of glycolysis (Fig. 4a) [38], while GSK inhibits the dehydrogenase A (LDHA), which converts pyruvate to lactate. First, we optimized the concentration of the drugs used so that cell physiology was not affected. Treatment of CRMM1, CRMM2 and 4T1 cells for 24 h with both inhibitors did not influence proliferation or cellular ATP production (Fig. 4b and c, Supplementary Fig. 3b and c). Importantly, under these conditions, the secretion of lactate from the highly glycolytic 4T1 cells and the CRMM1 and CRMM2 cells was significantly reduced to the level of the 67NR non-glycolytic cells, indicating that CoM cells indeed secrete lactate at a level comparable to 4T1 cells (Fig. 4d, Supplementary Fig. 3d).

Subsequently, we evaluated the metabolic properties of 67NR, 4T1, CRMM1 and CRMM2 after xenografting. To do so, we dissected zebrafish xenografts and analyzed the expression of glycolytic enzymes in the cancer cells via qPCR, including HK1; phosphofructokinase 1 (PFK1), which is the rate limiting enzyme that converts fructose 6-phosphate to fructose 1–6 bisphosphate and has a vital role in leading endothelial cells (ECs) sprouting; pyruvate dehydrogenase kinase (PDK), which regulates the catalytic activity of pyruvate decarboxylation oxidation, and it further links glycolysis with the tricarboxylic acid cycle and ATP generation; and lactate dehydrogenase A (LDH-A), which controls the final formation and release of lactic acid. As expected, 4T1 expressed significantly higher levels of HK1, PFK1, PDK, and LDHA than 67NR (Fig. 4e). CRMM1 and CRMM2 engraftments showed similar high expression levels as the 4T1 reference. Therefore, we conclude that 4T1, CRMM1, and CRMM2 retained their high glycolytic properties in the zebrafish model.

In all, the consistency between zebrafish macrophage-dependent angiogenesis and glycolytic properties of engrafted cells suggests that the lactate secreted by these cells during aerobic glycolysis may indeed attract macrophages and induces their polarization towards a M2 phenotype, leading to the expression of high levels of pro-angiogenic factors and the induction of angiogenesis in zebrafish.

### Supernatant secreted by conjunctival melanoma cells polarizes human macrophages to M2-like and leads to higher expression of proangiogenic factors

**Fig. 5:**
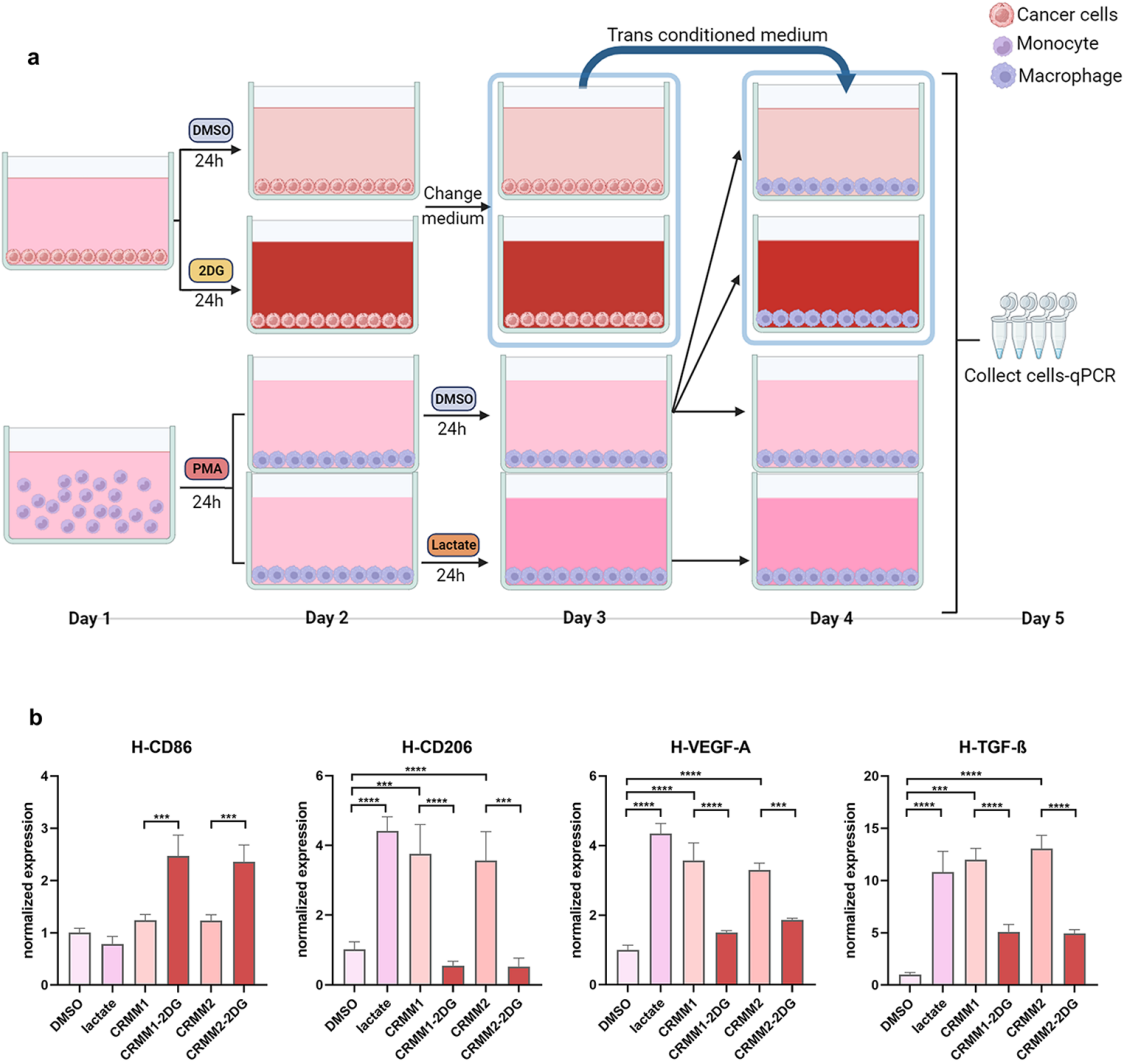
Supernatant secreted by CoM cells polarizes macrophages to an M2-like phenotype leading to higher expression of proangiogenic factors. **a** Schematic diagram of the conditioned medium experimental design. **b** Expression of CD86, CD206, VEGF-A, and TGF-β of macrophages in conditioned medium models

To further validate the translational relation between the metabolism of CoM cells and the polarization of macrophages towards their proangiogenic properties as observed in the zebrafish model, we exposed human macrophages derived from the THP-1 monocytic cell line [39] with the conditioned medium collected after 24 h treatment of CoM cells with DMSO solvent, 10 mM 2DG (Fig. 5a), or with 10µM GSK (Supplementary Fig. 4). As a positive control, the differentiated macrophages were directly treated with lactate. We then assessed the expression of polarization and pro-angiogenic gene markers (Fig. 5b). Lactate and the conditioned medium of CRMM1 and CRMM2 cells induced an increase in the expression of the M2 marker CD206 as well as of the pro-angiogenic markers VEGF-A and TGF-β which was ablated by inhibition of the glycolysis in the CoM culture with 2DG or GSK. Consistently, the expression of the M1 marker CD86 was only increased in the macrophages exposed to medium from treated CoM cells.

These results prove that glycolytic CoM cells are able to polarize human macrophages towards a M2 phenotype and increase their proangiogenic capacity.

### Inhibition of glycolysis attenuates macrophage-dependent angiogenesis induced by highly glycolytic CoM cells

**Fig. 6:**
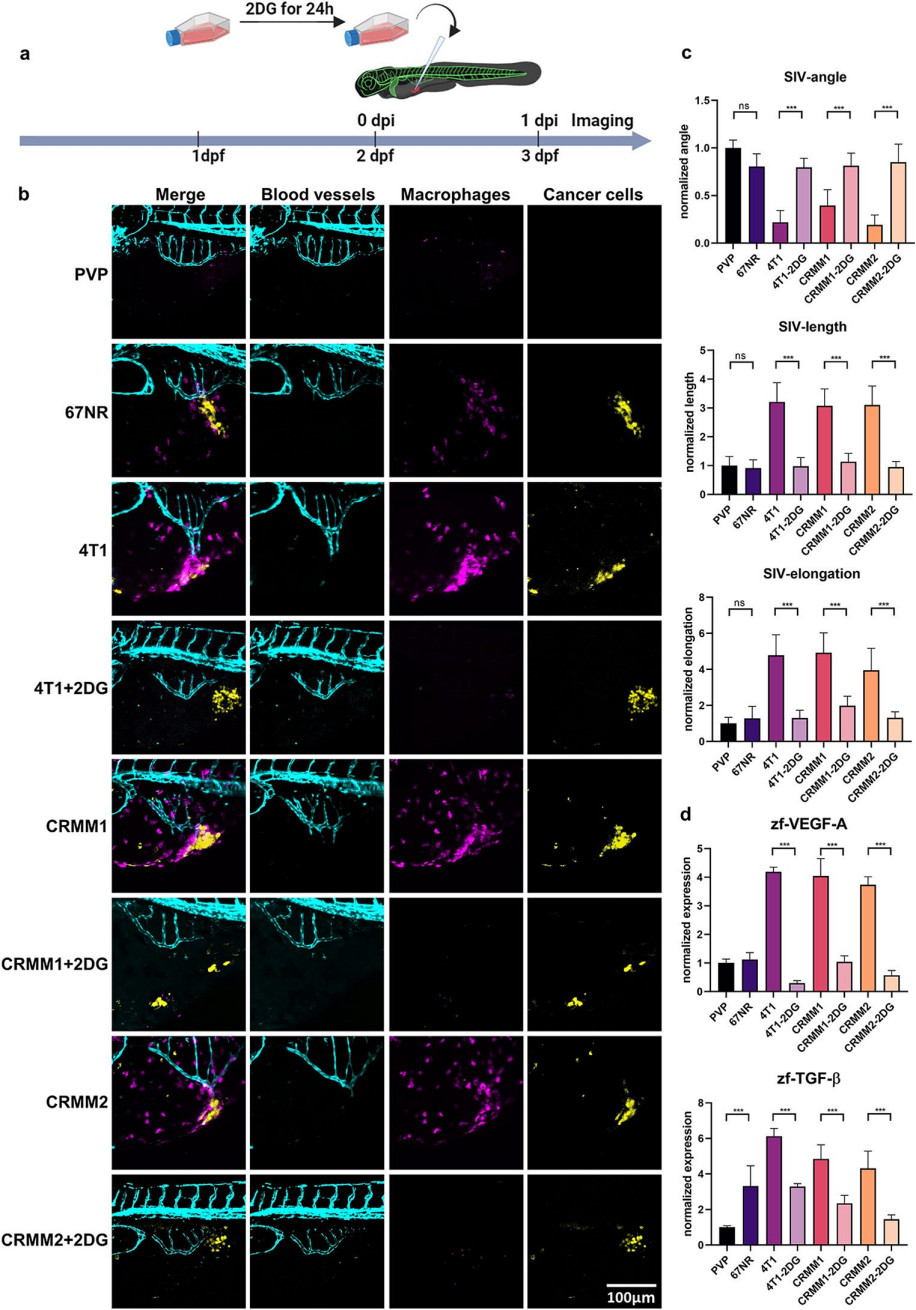
Inhibition of glycolysis attenuates macrophage-dependent angiogenesis of CoM cells. **a** Schematic diagram of 2DG treatment in cancer cells prior to their xenografting in zebrafish larvae. **b** Representative images of the angiogenesis response of embryos xenografted with CoM lines treated with and without 2DG treatment. **c** Quantification of the angiogenesis response in these conditions. **d** Quantification of the pro-angiogenic factors VEGF-A (*vegfaa*) and TGF-β (*tgfb1a*) in engrafted zebrafish

Since inhibition of glycolysis by 2DG reduced the production of VEGF-A and TGF-β in human macrophages in vitro, we aimed to validate this effect in vivo. Considering the lack of suitable rodent models for metastatic CoM, we employed the versatile zebrafish angiogenic assay previously presented. Cell lines 4T1, CRMM1, and CRMM2 were treated with 10 mM of 2DG for 24 h prior to their engraftment in the zebrafish PVS and imaged by confocal microscopy (Fig. 6a). As previously shown, the highly glycolytic tumors 4T1, CRMM1, and CRMM2 induced macrophage-dependent angiogenesis (Fig. 6b and c). However, treatment of these cells with 2DG before engraftment obstructed the sprouting of new vessels towards these xenografts. As a consequence of 2DG treatment, the SIV angle was flattened, its length and elongation were reduced (Fig. 6c). In contrast, the low glycolytic 67NR cells had similar phenotype as the control PVP.

Mechanistically, the 2DG treatment decreased the recruitment of zebrafish macrophages towards the engrafted 4T1, CRMM1, and CRMM2 cells as quantified by fluorescence intensity and by its expression marker, matrix metalloprotease 9 (mmp9) (Supplementary Fig. 5). In addition, zebrafish engrafted with untreated and 2DG treated cells were dissected and subjected to expression analysis of pro-angiogenic genes. As shown in Fig. 6d, the high expression of proangiogenic genes like VEGF-A and TGF-β induced by highly glycolytic cells was inhibited by 2DG treatment to the levels caused by engraftment of the low glycolytic 67NR cells. Therefore, inhibition of glycolysis in engrafted CoM cells by 2DG suppressed macrophage recruitment, as well as the M2 polarization responsible for secretion of proangiogenic factors in the zebrafish tumor microenvironment.

These results confirm that lactate secreted by glycolytic CoM cells attracts and polarizes macrophages to drive angiogenesis in the zebrafish model.

## Discussion

The mechanism of metastatic dissemination of conjunctival melanoma is poorly studied. This is mainly due to the rarity of CoM and therefore a lack of patient material and animal models resembling the metastatic stage of this disease [40]. The majority of studies are conducted in vitro or with small cohorts of primary CoM, making it impossible to visualize dynamic interactions between CoM cells and their microenvironment [41]. It is already known how the malignant and metastatic behavior of cutaneous melanoma, a disease with high genetic similarity, is often linked to increased angiogenesis, with abundant infiltrating TAMs [42]. We used the zebrafish xenograft model, a powerful platform to investigate dynamic cellular interactions within the TME, due to its transparency [43]. Injecting fluorescent CoM cells into transgenic embryos with fluorescently-labeled macrophages and vessels allowed us to study their interactions and behavior [44]. We elucidated the functional significance of macrophages in the neo-angiogenesis process induced by CoM xenografts. We found that lactate produced by the highly glycolytic CoM cells attracts macrophages and polarizes them into a pro-tumoral phenotype leading to secretion of angiogenic cytokines to further stimulate macrophage-dependent angiogenesis. Consequently, inhibition of glycolysis in CoM cells or depletion of macrophages attenuated the angiogenetic response. Therefore, we concluded that CoM cells with a high glycolytic index may use TAM-mediated angiogenesis for their metastatic spread.

Initially, we assessed if engrafted CoM cells are able to induce sprouting or attract blood vessels from the developing SIV. To do so, we used microinjection to inject CoM cells in the PVS space, a process that creates a piercing wound and is accompanied by the recruitment of macrophages. Indeed, through Time Lapse confocal imaging, we observed how injection of the PVP solvent attracted macrophages at 2hpi that started the wound repair and resolved the inflammatory process within 6hpi without affecting SIV development. On contrast, we observed strong continued macrophage recruitment and a gradual angiogenic response towards the injection foci within 24 hpi after grafting CoM cells. Inflammatory responses associated with wound healing and tumors are remarkably similar: tumors have been found to behave as wounds that do not heal, in order to establish a favorable microenvironment [45]. Accordingly, in the presence of CoM cells, no resolution phase was observed and macrophages continued to accumulate close to engrafted cells until 24hpi. These results suggest that the engrafted CoM cells continuously induce an inflammatory response, thereby recruiting macrophages. This is consistent with a previous immunofluorescent analysis of 27 primary CoM patient samples that demonstrated infiltrated macrophages in varying amounts, despite that the number of pro-tumoral macrophages did not relate significantly to survival or recurrence of CoM [46].

By chemically ablating macrophages after engraftment, we were able to prove that in the zebrafish model, macrophages recruited to engrafted CoM cells polarized towards an M2-like type and produced pro-angiogenic factors like VEGF-A, TGF-β and IL-10 to support the angiogenic response. The limitation of our approach is that we depleted the entire embryo macrophage population [47]. Interestingly, VEGF immunoreactivity was previously detected in primary CoM tissues [10], as well as in the two CoM cell lines (CRMM1, CRMM2) derived from a local recurrence that we used in this study, which affected migration and proliferation of endothelial cells [46]. In our model we cannot fully exclude the contribution to angiogenesis of endogenous VEGF produced by the CoM cells themselves. However, as depletion of the macrophages significantly reduced zf-VEGF in the CoM xenograft model and blocked angiogenesis, we suggest that tumor-associated macrophages are the main source of VEGF to initiate the angiogenic response in this model. This assumption is in line with previously published results concerning lung carcinoma, cutaneous melanoma and colon carcinoma xenograft models in mice [25].

To address how CoM cells attract macrophages to promote the neo-angiogenic response in zebrafish we decided to assess the importance of lactate as a well-defined factor that polarizes macrophages to a tumor-promoting stage [48]. One of the best-known processes in cancer is the Warburg effect, by which cancer cells perform lactate fermentation in the presence of oxygen and secrete lactate. The recent discovery of frequent somatic mutations in the new candidate oncogene *ACSS3*, one of the three genes encoding acetyl-CoA synthetase proteins, catalyzing the synthesis of acetyl-CoA from acetate in an ATP-dependent manner, supports a possible relationship between CoM tumorigenesis and the Warburg effect [27]. However, it was still unknown if zebrafish macrophages could sense lactate as reported for mammalian macrophages, or if CoM cells were highly glycolytic in vitro or in vivo.

First, we used a zebrafish macrophage attraction assay to test whether lactate functions as a chemoattractant to recruit macrophages, comparing results to a positive control, human CCL2 [49]. Further, we demonstrated in the zebrafish xenograft model that highly metastatic, glycolytic cell lines, such as 4T1, indeed caused a significant angiogenic response and recruited more macrophages compared to the PVP control and their isogenic but low glycolytic 67NR cells [30]. We then asked if CRMM1 and CRMM2 cells could also secrete lactate to drive macrophage recruitment and polarization, and lead to induction of angiogenesis in the zebrafish model. Considering that there are no records describing the metabolic index of CoM cells, we directly measured the level of lactate in their supernatant without and with pre-treatment with the glycolysis inhibitor 2DG, and compared their lactate levels to the reference breast cancer lines 4T1 and 67NR. The secretion levels of lactate from CRMM1 and CRMM2 cells were comparable to 4T1 cells and were reduced by 2DG to the level of 67NR non-glycolytic cells. In addition, the qPCR results also revealed that 4T1, CRMM1 and CRMM2 engraftments expressed significantly higher levels of glycolytic enzymes than 67NR xenografts, indicating that 4T1, CRMM1, and CRMM2 maintained high glycolytic properties in zebrafish xenografts. Thus, we concluded that zebrafish macrophages responded to lactate as described for mammalian macrophages and that CoM cells were performing glycolysis in vitro and in vivo.

We then inhibited the glycolysis of CoM cells using 2DG and transferred their supernatant to cultured human macrophages, which reduced their production of VEGF-A and TGF-β. We finally validated this effect in vivo using a zebrafish angiogenic assay. The results demonstrated that treatment of 4T1, CRMM1, and CRMM2 with 2DG before engraftment stopped the sprouting of new vessels towards the grafted cells. Mechanistically, the inhibition of glycolysis in engrafted CoM cells by 2DG treatment suppressed macrophage recruitment and their M2 polarization, responsible for delivery of proangiogenic factors in the zebrafish TME. These results confirm that lactate secreted by glycolytic conjunctival melanoma cells attracts and polarizes macrophages to drive angiogenesis in the zebrafish model. Importantly, given that our study involved the polarization of human macrophages to an M2-like type, which led to a higher expression of proangiogenic factors, we believe that the mechanisms that we discovered in the zebrafish model can be extrapolated to the human situation with human macrophages [50]. Unfortunately, our short-time angiogenic model cannot predict if macrophage-dependent angiogenesis can lead to metastatic dissemination and development of metastatic CoM. To test this, more suitable metastatic rodent models need to be generated.

In conclusion, this study has aided in the understanding of the interactions between macrophages and CoM cells, and serves as a foundation for further pre-clinical proof that macrophages induce neo-angiogenesis in CoM melanoma with a high glycolytic index as a possible mechanism of metastatic dissemination.

## Electronic supplementary material

Below is the link to the electronic supplementary material.

Supplementary Material 1

Supplementary Material 2

## Data Availability

All data generated or analyzed during this study are included in this paper and its supplementary information files.
