# Supplementary material for: Lactate secreted by glycolytic conjunctival melanoma cells attracts and polarizes macrophages to drive angiogenesis in zebrafish xenografts
Source: Angiogenesis. 2024 Jun 6;27(4):703–17. doi: 10.1007/s10456-024-09930-y (PMC11564320; doi:10.1007/s10456-024-09930-y)
Supplement: Supplementary file 2 — Supplementary Material 2 [file 10456_2024_9930_MOESM2_ESM.docx]

**Supplementary Information**


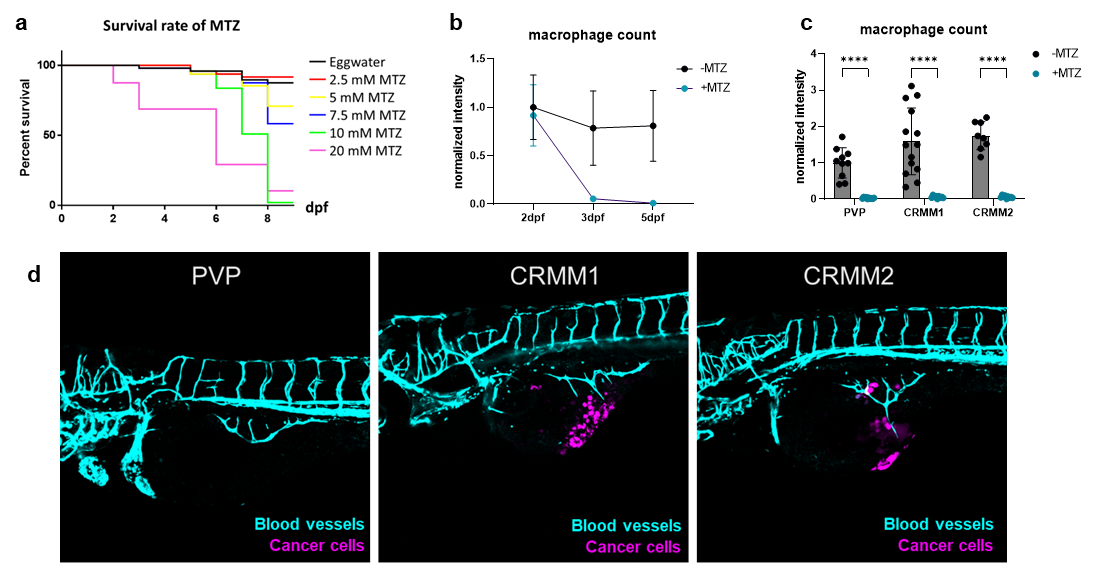


**Supplementary Fig. 1 Toxicity and effect of metronidazole (MTZ) in ablating macrophages in live zebrafish larvae.** **a** Kaplan-Meier curve of the survival rate of Tg(*kdrl*:EGFP^s843^; *mpeg1*:GAL4-VP16^gl24^; UAS-E1b:NfsB-mCherry^i149^) larvae treated with increasing concentrations of MTZ. **b** Efficiency of 2.5 mM of MTZ in depleting macrophages from 2dpf to 5dpf. **c** Fluorescence intensity of the macrophage channel in zebrafish xenografted with tumors at 5dpf with and without 2.5 mM MTZ. **d** Tg(*kdrl*:EGFP^s843^) larvae xenografted in the PVS with CRMM1 and CRMM2 and treated with 2.5mM of MTZ.

**
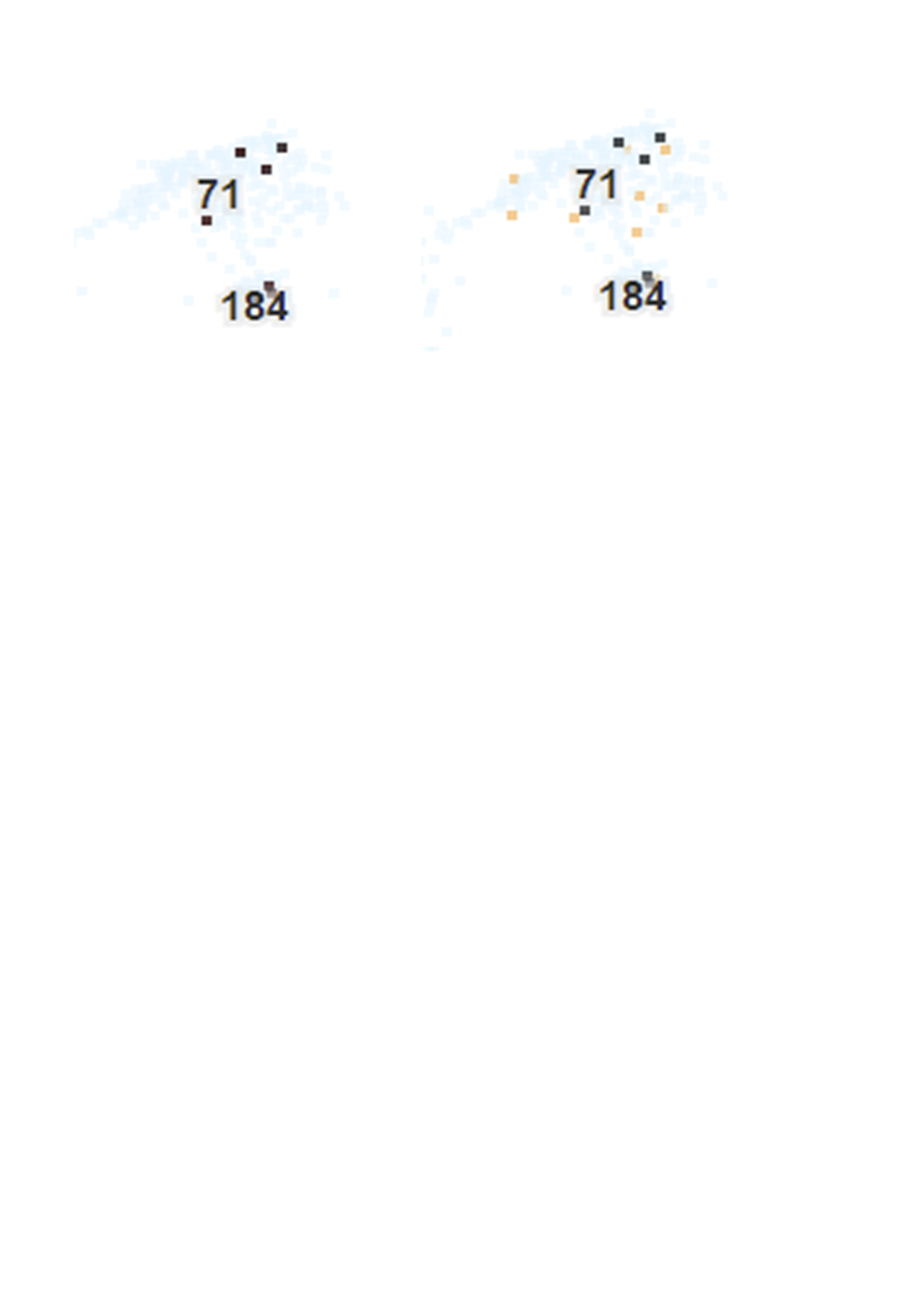
**

**Supplementary Fig. 2 Lactate receptors are present in the zebrafish macrophage population**. Expression intensity of the genes *Gpr132b* (left) and *hcar1-3* (right) in the cell clusters 71 and 184 from the Miller lab single cell RNAseq, which are annotated as macrophages


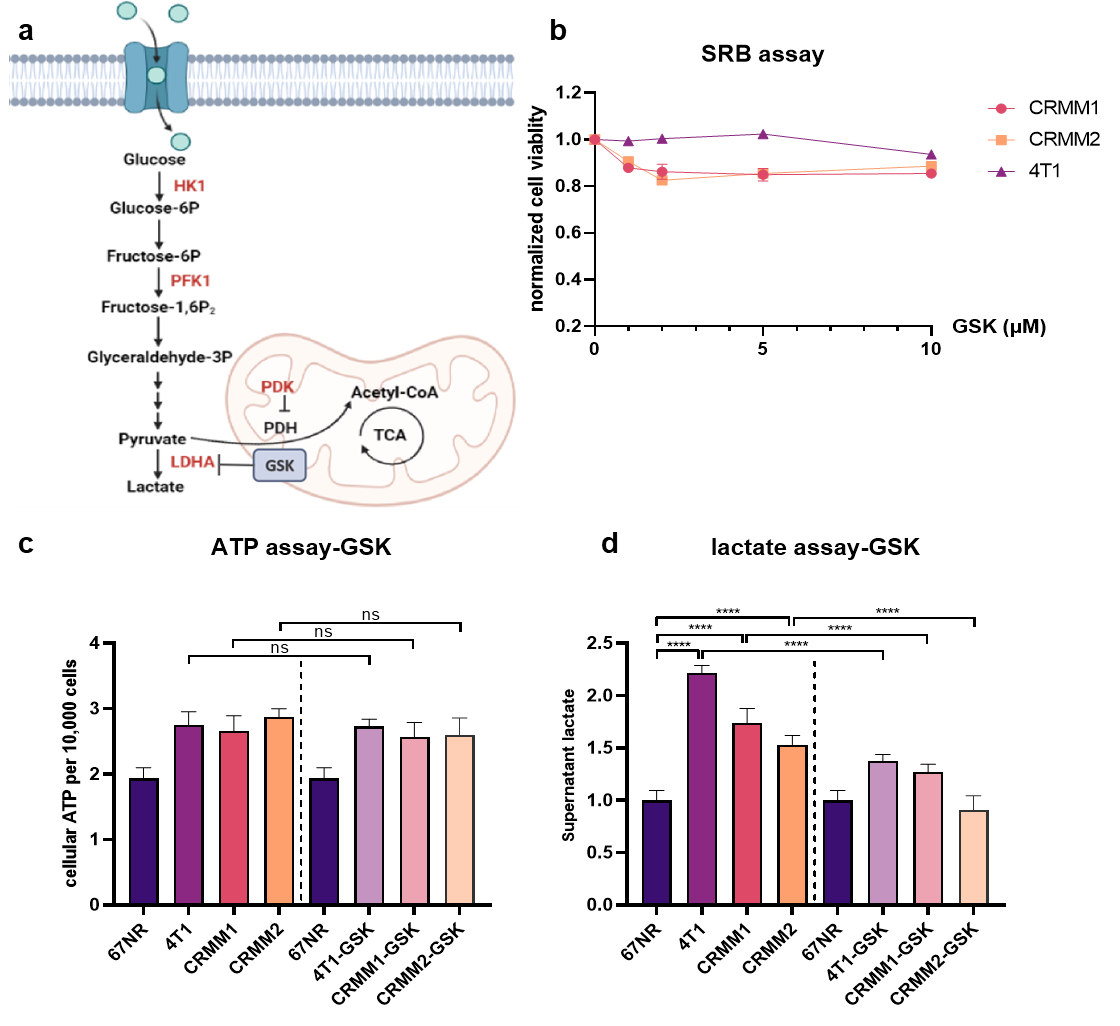


**Supplementary Fig. 3 Toxicity and effect assays of the lactate inhibitor GSK**. **a** Schematic representation of the glycolysis pathway with key enzymes in the process, showing the inhibition of GSK on the lactate dehydrogenase A (LDHA). **b** Quantification of cell viability during treatment with increasing concentrations of GSK for 24 hrs. **c** Cellular ATP levels of 67NR and 4T1, CRMM1, and CRMM2 after treatment with 10μM GSK. **d** GSK inhibited the lactate production in 4T1, CRMM1, and CRMM2 cells.


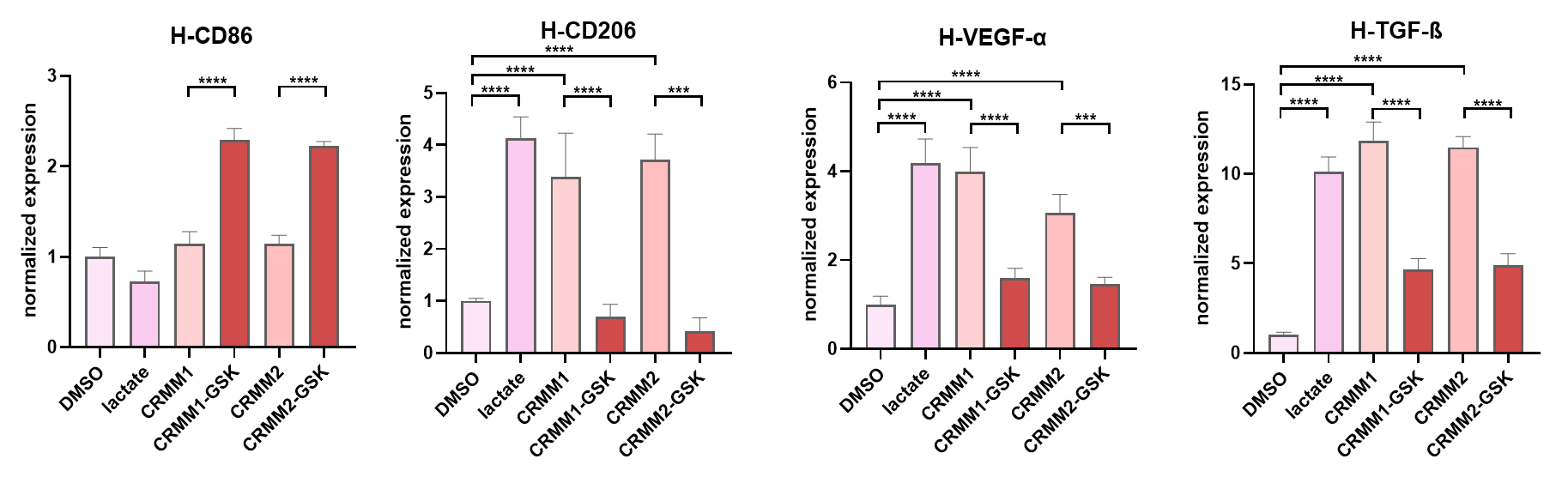


**Supplementary Fig. 4** Expression of CD86, CD206, VEGF-A, and TGF-β of macrophages in conditioned medium from CRMM1 cells treated with or without 10μM GSK for 24h.

**
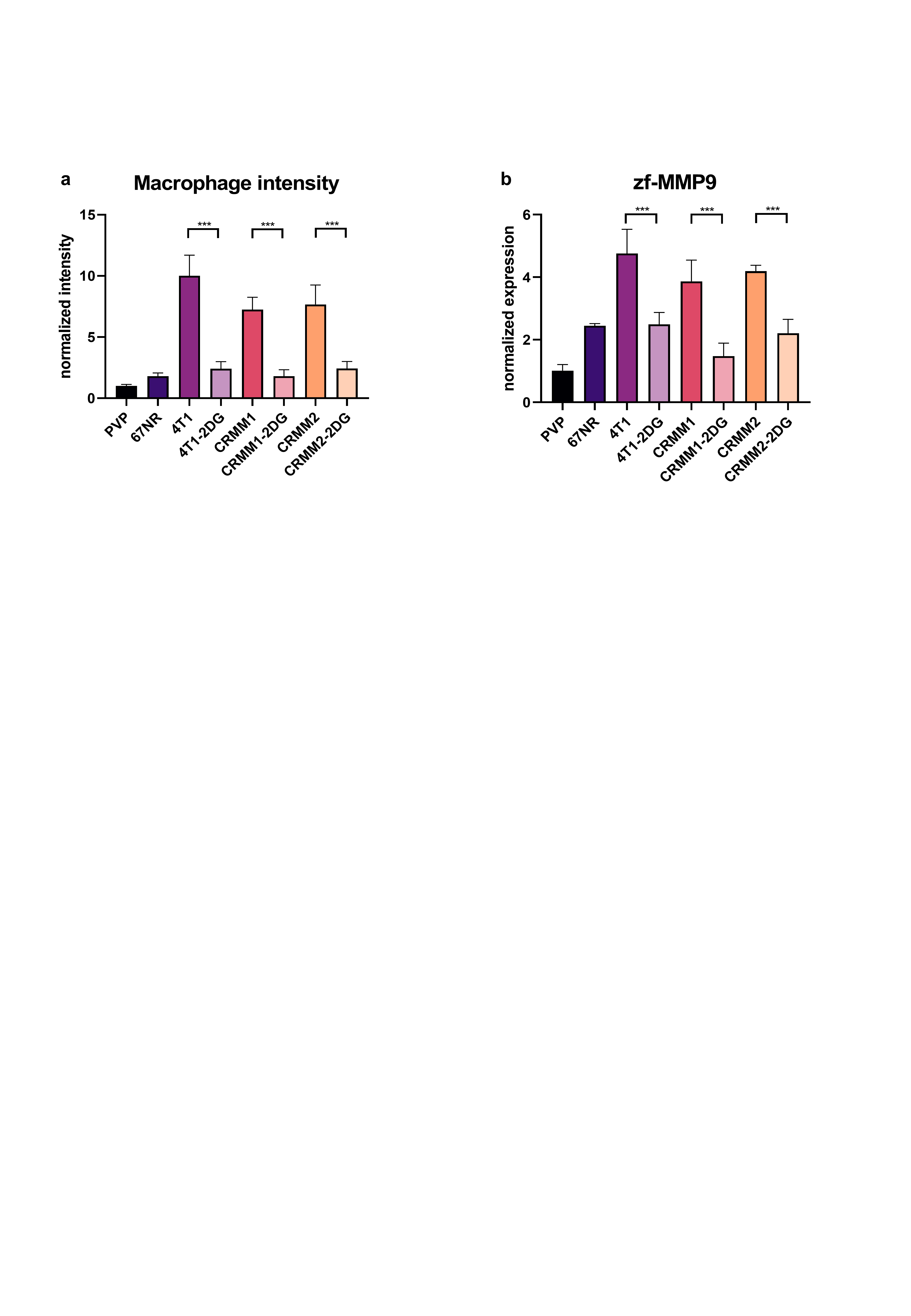
**

**Supplementary Fig. 5 Macrophage recruitment capacity was inhibited by pretreatment of cancer cells with 2DG.** **a** Macrophage channel fluorescence intensity in the tumor site. **b** MMP9 expression in the TME of engrafted zebrafish.

**Supplementary Movie 1 Timelapse of SIV angiogenesis assay in CoM-xenografted zebrafish.** Zebrafish larvae with fluorescent vessels (cyan) and macrophages (magenta) were xenografted with CRMM2 cells (yellow) in PVS, anaesthetized and imaged using a Leica TCS SP8 confocal microscope with a 20X objective equipped with 488-nm, 532-nm, and 638-nm laser lines for 24h.

**Supplementary Table 1 Percentages of identity between zebrafish and human gpr132 and hcar1-3 genes**

| **Receptor name** | **Sequence level** | **Percentage of identity** |
| --- | --- | --- |
| GPR132 G protein-coupled receptor 132 | DNA | 68.78% |
| GPR132 G protein-coupled receptor 132 | mRNA | 68.78% |
| GPR132 G protein-coupled receptor 132 | protein | 42.75% |
| hcar1-3 hydroxycarboxylic acid receptor 1-3 | DNA | 73.02% |
| hcar1-3 hydroxycarboxylic acid receptor 1-3 | mRNA | 73.02% |
| hcar1-3 hydroxycarboxylic acid receptor 1-3 | protein | 39.80% |

**Supplementary Table 2 Sequences of primers used for RT-qPCR reactions**

| **Gene** | **Forward** | **Reverse** |
| --- | --- | --- |
| ***Human*** |  |  |
| *β-actin* | GCGAGAAGATGACCCAGAT | GAGGCGTACAGGGATAGC |
| *HK1* | CTGCTGGTGAAAATCCGTAGTGG | GTCCAAGAAGTCAGAGATGCAGG |
| *PFK1* | GCTTCTAGCTCATGTCAGACCC | CCAATCCTCACAGTGGAGCGAA |
| *PDK* | CATGTCACGCTGGGTAATGAGG | CTCAACACGAGGTCTTGGTGCA |
| *LDHA* | GGATCTCCAACATGGCAGCCTT | AGACGGCTTTCTCCCTCTTGCT |
| *CD86* | CCATCAGCTTGTCTGTTTCATTCC | GCTGTAATCCAAGGAATGTGGTC |
| *CD206* | AGCCAACACCAGCTCCTCAAGA | CAAAACGCTCGCGCATTGTCCA |
| *VEGF-α* | TTGCCTTGCTGCTCTACCTCCA | GATGGCAGTAGCTGCGCTGATA |
| *TGF-β* | TACCTGAACCCGTGTTGCTCTC | GTTGCTGAGGTATCGCCAGGAA |
| ***Zebrafish*** |  |  |
| *tbp* | CGGTGGATCCTGCGAATTA | TGACAGGTTATGAAGCAAAACAACA |
| *vegfaa* | CTCCTCCATCTGTCTGCTGTAAAG | CTCTCTGAGCAAGGCTCACAG |
| *tnfa* | ACCAGGCCTTTTCTTCAGGT | TTTGCCTCCGTAGGATTCAG |
| *il4* | GCACTGTATTCGTCTCGGGTTTTA | TTTTCCCCAGATCTACAAGGAAGA |
| *il1b* | TGTGTGTTTGGGAATCTCCA | CTGATAAACCAACCGGGACA |
| *tgfb1a* | TCTGGGAACTCGCTTTGTCTCCAA | TCTTCTGAACCCTGCAGCCATTCT |
| *il10* | CTTTAAAGCACTCCACAACCCCAA | CTTGCATTTCACCATATCCCGCTT |
| *nos2a* | CATTGATCTCCGTGACAGCC | CATGCTACTGGAGGTGGGTG |
| *mmp9* | CATTAAAGATGCCCTGATGTATCC | AGTGGTGGTCCGTGGTTGAG |
